# Solid lubricant behavior of MoS_2_ and WSe_2_-based nanocomposite coatings

**DOI:** 10.1080/14686996.2016.1275784

**Published:** 2017-03-01

**Authors:** Santiago Domínguez-Meister, Teresa Cristina Rojas, Marta Brizuela, Juan Carlos Sánchez-López

**Affiliations:** ^a^ Instituto de Ciencia de Materiales de Sevilla, CSIC-Univ. de Sevilla, Seville, Spain; ^b^ TECNALIA, Donostia-San Sebastián, Spain

**Keywords:** Functionally graded material, Raman spectroscopy, analytical electron microscopy, structure–property relationship, friction, 10 Engineering and Structural materials, 102 Porous / Nanoporous / Nanostructured materials, 105 Low-Dimension (1D/2D) materials, 306 Thin film / Coatings, 503 TEM, STEM, SEM, 501 Chemical analyses

## Abstract

Tribological coatings made of MoS_2_ and WSe_2_ phases and their corresponding combinations with tungsten carbide (WC) were prepared by non-reactive magnetron sputtering of individual targets of similar composition. A comparative tribological analysis of these multiphase coatings was done in both ambient air (30–40% relative humidity, RH) and dry nitrogen (RH<7%) environments using the same tribometer and testing conditions. A nanostructural study using advanced transmission electron microscopy of the initial coatings and examination of the counterfaces after the friction test using different analytical tools helped to elucidate what governs the tribological behavior for each type of environment. This allowed conclusions to be made about the influence of the coating microstructure and composition on the tribological response. The best performance obtained with a WSe_x_ film (specific wear rate of 2 × 10^−8^ mm^3^ N^–1^m^–1^ and a friction coefficient of 0.03–0.05) was compared with that of the well-established MoS_2_ lubricant material.

## Graphical abstract

**Figure:**
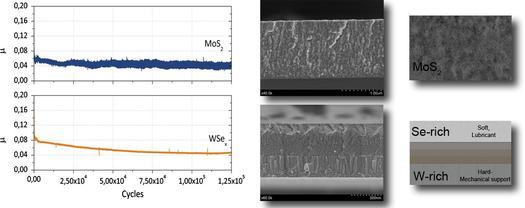

## 1. Introduction

The interaction between surfaces in mechanical contacts significantly affects energy loss, machinery performance and endurance. Introducing a lubricant reduces friction and wear, which in turn positively affects the fuel consumption, maintainability and lifetime costs of engineering components. The load-bearing capacity and lubricity of the surfaces are substantially improved if the solid lubricant film can shear easily. The advantage of solid lubricants in vacuum and aerospace applications is that they can substitute for oil lubricants and function at high temperatures or in vacuum. Typical examples of dry lubricant films are soft metals (such as Ag, Au, In, Sn, and Pb), layered inorganic compounds (such as MoS_2_, graphite, and hexagonal boron nitride) and polymers such as Teflon [1–3]. MoS_2_ has been the most commonly employed intrinsic solid lubricant over the past 40 years [4–6] and still is the object of exhaustive studies for determining its functionality for space applications [7–9]. However, these coatings are soft, have poor adhesion and load-bearing capacity, and degrade in humid air [10], although not as significantly as graphite. Sputtered MoS_2_ films display friction coefficients from 0.005 to 0.05 [11,12] and wear rate of the order of 10^−8^ mm^3^ N^–1^m^–1^ when operating in vacuum or inert atmosphere. In humid air, the friction coefficient increases to 0.15–0.30, and the wear resistance decreases by a factor of 10 to 10^3^ with respect to vacuum, depending on the film properties. In humid air, dangling or unsaturated bonds on the edges of the basal planes react with moisture and oxygen in the environment, leading to higher friction and eventual thin film failure. However, recent studies of Colas et al. [7,13] showed that the wear life of MoS_2_ can be extended if a reasonable amount of contaminants is present in both the coatings and the environment.

Likewise, the disulfides or diselenides of certain transition metals, principally WS_2_, NbSe_2_, MoSe_2_ and WSe_2_, are recognized for their lubricant properties but also exhibit low load carrying capacity. All of them have a low shear strength (τ = 1–2 MPa) [14] when working in clean environments (i.e. inert gas or vacuum). The origin of the low friction lies in their anisotropic layered structure, that is, covalent bonding within the adjacent lamellae and weak Van der Waals forces between them. During sliding contact, the basal planes are oriented parallel to the surface in contact so that (002)-oriented transfer layers are generated during the steady state [3]. The parallel basal planes slide over one another by inter and intra-crystalline slip, providing easy shear. The sensitivity to humid air is reported to be less in the case of diselenides [14–18], which is of particular interest for space components that require testing or storage periods on Earth.

Increased wear resistance and load bearing capacity of metal chalcogenides have been reported by alloying with metals (Ti, Au, Pb, Ni, Cr) [19–21] or non-metals (N or carbon) [22,23]. The strength and oxidation resistance can also be improved by designing and using smart tribological coating architectures such as nanocomposite (MoS_2_/WC [24], WS_2_/WC/DLC [25], TiN/MoS_x_ [26], TiCrBN/WSe_x_ [27], WS_2_/ZnO [28], TiSiN/WS_2_ [29]) and/or nanostructured coatings (WSe_x_/a-W(Se) [18], metal/MoS_2_ [30], MoS_2_/WS_2_ [31]), providing a longer lifetime and enhanced performance under variable working conditions [32,33]. Comparison of the tribological responses of different solid lubricants is a challenging task since their response depends on the sum of factors: type of dichalcogenide, nanostructure, architecture (alloyed, nanocomposite or multilayer), type of test (reciprocating, rotative, fretting, etc.) and measurement conditions (applied load, nature of surrounding atmosphere, temperature, etc.). Moreover, in most cases, the papers placed the emphasis on improving the numeric values of the friction coefficient and the specific wear rates rather than investigating the reasons behind such tribological performance.

Metal selenides thin films have been less studied than sulfides for tribological purposes. Among them, tungsten selenide coatings have been mostly prepared by pulsed laser [34–36] deposition, while magnetron sputtering was used in combination with C and N [14,16,23,25,32] or with metals [37]. In our previous work, optimized engineered tribological coatings based on MoS_2_ and WSe_2_ were prepared by non-reactive magnetron sputtering [17,18,24]. Codeposition with WC targets was tried with the aim of improving the wear and oxidation resistance of the films, particularly in ambient air. Previous attempts to reinforce the tribological properties used sputtering metals, graphite or inserting carbon pellets in the chalcogenide target. The influence of carbon and oxygen on the tribological behavior of these compounds is still unclear, and careful investigation of the contact surfaces of the composite layers under different atmospheres is still needed.

In this work, we make a comparative tribological analysis of these multiphase coatings both in ambient air and dry nitrogen environments using the same tribometer and testing conditions. This series of tests would allow direct comparison of the performance of these tailored solid lubricant coatings with the well-known MoS_2_ material (perhaps the most employed solid lubricant material for vacuum and space applications) under the same tribological conditions. A nanostructural study by advanced transmission electron microscopy of the initial coatings and examination of the counterfaces by different analytical tools after the friction test will enable an understanding of what governs the tribological behavior for each type of environment. This will allow conclusions to be made about the influence of the coating microstructure and composition on the tribological response.

## 2. Materials and methods

A CemeCon CC800/8 magnetron sputtering unit (Aachen, Germany) was employed for the deposition of the MoS_2_ and alloyed MoS_2_-WC films from rectangular targets (200 mm × 88 mm × 5 mm) of MoS_2_ and WC in an Ar discharge at a pressure of (5‒7) × 10^−3^ mbar. The WSe_x_ coatings were prepared by radio frequency (r.f.) magnetron sputtering at 13.56 MHz in an Ar atmosphere (5 × 10^−3^ mbar) of WSe_2_ and WC targets (50.8 mm in diameter). A modulated pulsed bias was applied during synthesis. The pulse conditions were set at a frequency of 250 kHz, a duration of 1616 ns (60% duty cycle) and 25 W of power. Further details about film deposition can be found elsewhere [17,24].

The chemical composition of the films was determined by electron probe microanalysis (EPMA). The EPMA equipment was a JEOL JXA-8200 SuperProbe instrument equipped with four wavelengths (WDS) and one energy-dispersive X-ray (EDX) detector (Jeol Ltd., Tokyo, Japan). The morphology and thickness of the coatings were investigated by cross-sectioning the specimens and subsequent observation by scanning electron microscopy (SEM) in a high resolution field emission gun microscope, Hitachi-4800 (Tokyo, Japan), equipped with an EDX detector (Bruker, XFlash4100, Billerrica, MA, USA). The structure and phase composition were studied by X-ray diffraction (XRD) and Raman spectroscopy. The XRD patterns were obtained with a Siemens D5000 diffractometer (Billerrica, MA, USA) in the conventional θ-2θ° Bragg–Brentano configuration and a grazing incidence angles of 1° and 5° using Cu Kα radiation. Raman spectra measurements (150–2000 cm^−1^) were carried out with a LabRAM (Horiba Jobin Yvon, Palaiseau, France) spectrometer equipped with a true confocal microscope, a charge-coupled device detector and a solid-state laser (532 nm) operated at 2 mW to avoid damaging the sample. All the samples were analyzed with 100 s exposure times and aperture openings of 100 μm using 50 × magnification.

The mechanical properties were measured with a Fischerscope H100 dynamic microprobe instrument (Fischer Instruments, Barcelona, Spain) using a conventional Vickers indenter at loads up to 10 mN. The maximum load was selected in such a way that the maximum indentation depth did not exceed 10–15% of the coating thickness in order to avoid the influence of the substrate. Tribological tests have been carried out in a CSM tribometer (Antoon Paar, Graz, Austria) using a 6 mm 100Cr6 ball in linear and rotating configurations under atmospheric pressure and dry nitrogen environments. Reciprocating tests were done at 2 N of applied load (maximum contact pressure of ~0.83 GPa), stroke length of 2 mm, and 2 mm s^–1^ of linear speed during 2500 cycles. Long duration tests were performed at 5 cm s^–1^ of sliding speed and 2 N of applied load during 125,000 cycles. Examination of the wear tracks and ball scars after concluding the tribological tests were done using optical microscopy. For the advanced microstructural characterization and elucidation of the friction mechanism, cross-sectional specimens were prepared by conventional procedures and by focused ion beam (FIB), and studied using an FEI Tecnai G2 F30 S-Twin high resolution transmission electron microscope (HRTEM) operated at 300 kV, with 0.2 nm point resolution (Eindhoven, The Netherlands), equipped with a high-angle annular dark field (HAADF) detector from Fischione Instruments (Roskilde, Denmark), an X-Max EDX detector from Oxford Instruments (Abingdon, Oxfordshire, UK), and a Quantum 96 image filter from Gatan (Evry, France). Digital micrograph software from Gatan was used to acquire images and perform further image and electron energy-loss spectroscopy (EELS) analysis. Tecnai Imaging and Analysis (TIA) software from FEI company (Eindhoven, The Netherlands) was used for EDX spectra quantification.

## 3. Results and discussion

### 3.1. Morphological and chemical characterization

The chemical composition of the coatings as determined by EPMA is summarized in Table 1. It can be noticed that the Se/W ratios do not reach the stoichiometric value of 2, in contrast to the MoS_2_ films. This depletion in selenium has been already highlighted in our previous publications and for other chalcogenides prepared by PVD techniques. Carbon and oxygen contamination is present at the level of 5–10 at.% due to the residual vacuum and air exposure. The thicknesses of the coatings range between 0.9 and 1.8 μm. The film hardness is in the range of 4–7 GPa with corresponding Young modulus values varying between 75 and 100 GPa. This represents a significant enhancement over the bulk values for this type of material, which are typically below 0.5 GPa [31,38] thanks to their nanostructure and tailored chemical composition.

**Table 1.** Chemical composition obtained from EPMA analysis, film thickness and hardness values for the set of coatings.

	Mo	S or Se	W	C	O	Thickness (μm)	Hardness (GPa)	Elasticmodulus (GPa)
MoS_2_	28.6	56.1	‒	8.0	7.4	1.4	4.0	75
MoS_2_-WC	25.8	49.8	5.9	11.3	7.2	1.8	6.9	99
WSe_x_	‒	44.7	41.7	5.5	8.1	0.9	5.1	90
WSe_x_-WC	‒	31.6	48.4	11.3	8.7	1.8	4.6	70

**Figure 1.**
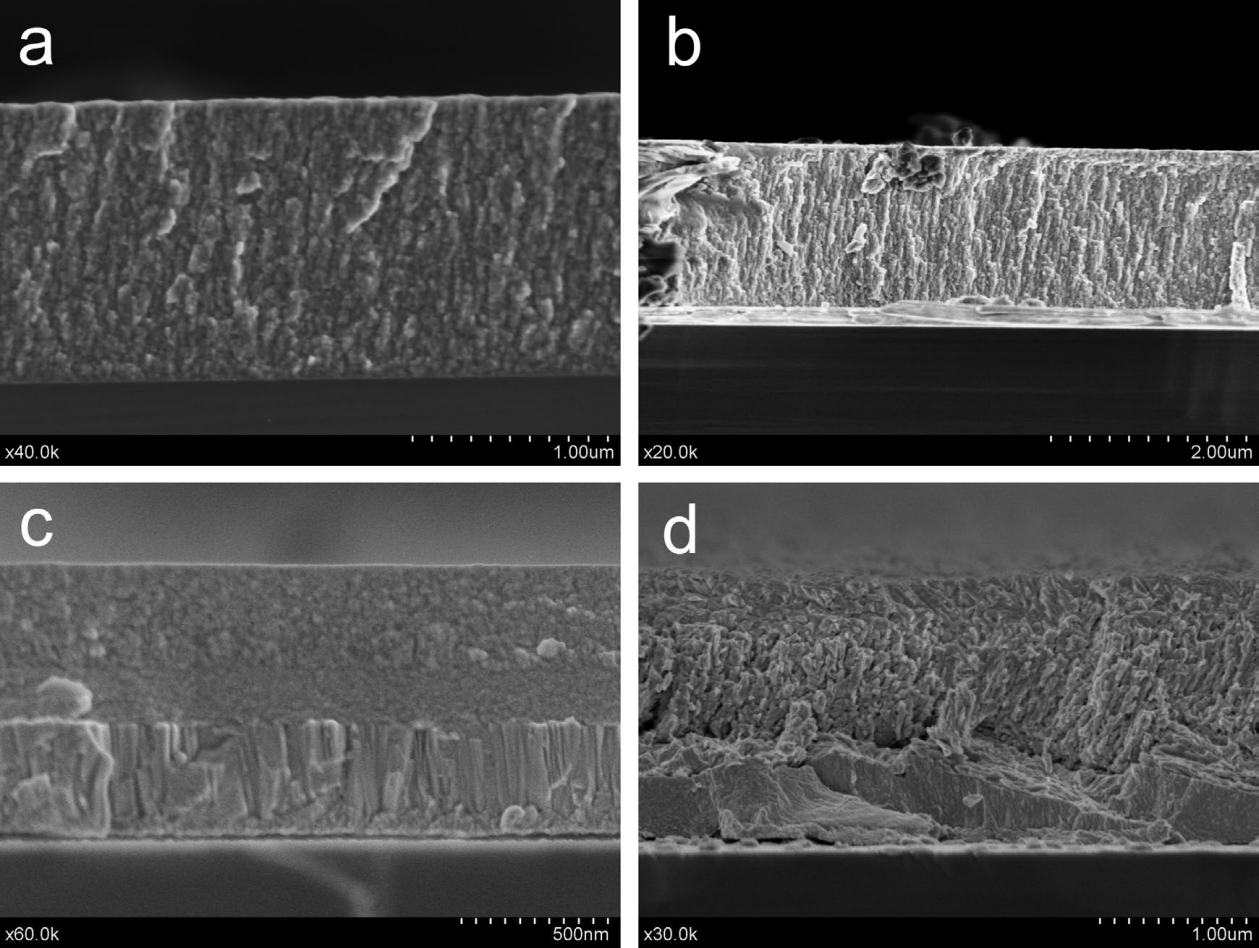
SEM cross-sectional views of the MoS_2_ and WSe_2_-based coatings: (a) MoS_2_, (b) MoS_2_-WC, (c) WSe_x_, (d) WSe_x_-WC.

The film microstructure was studied by scanning electron microscopy performed on cross-sectional specimens. Transition metal dichalcogenides develop generally columnar morphologies, as demonstrated here in Figure 1. The molybdenum disulfide coatings show a fine columnar microstructure (Figure 1(a) and 1(b)), but the MoS_2_ reference film appears more granular. Three different layers constitute the microstructure of the WSe_x_ film (Figure 1(c)), from highly columnar at the bottom to dense granular in the top. In the last picture corresponding to the WSe_x_/WC film (Figure 1(d)), a tilted columnar structure is grown onto a packed underlayer. The observed microstructure is partially due to the oxygen and carbon contamination. Oxygen, water and other reactive species (C_x_H_y_, CO_2_, H_2_) [13] can easily penetrate the film through the column and grain boundaries in transition metal chalcogenide thin films, contributing to the increased C and O contamination as reported by previous authors [14,38]. These species are preferentially adsorbed and trapped inside the coating without chemical reaction with the chalcogenide. The analysis by mass spectrometry of the desorbed gases during a friction test carried out in ultrahigh vacuum also demonstrated the desorption of these species [39].

**Figure 2.**
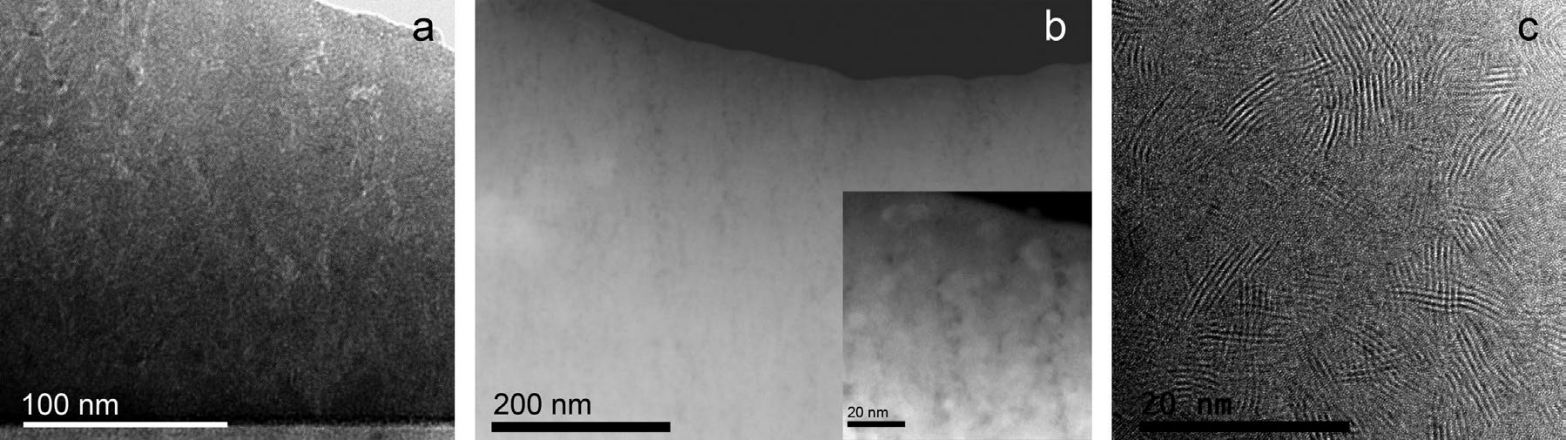
(a) TEM, (b) HAADF-STEM, and (c) HRTEM micrographs of the MoS_2_ film.

**Figure 3.**
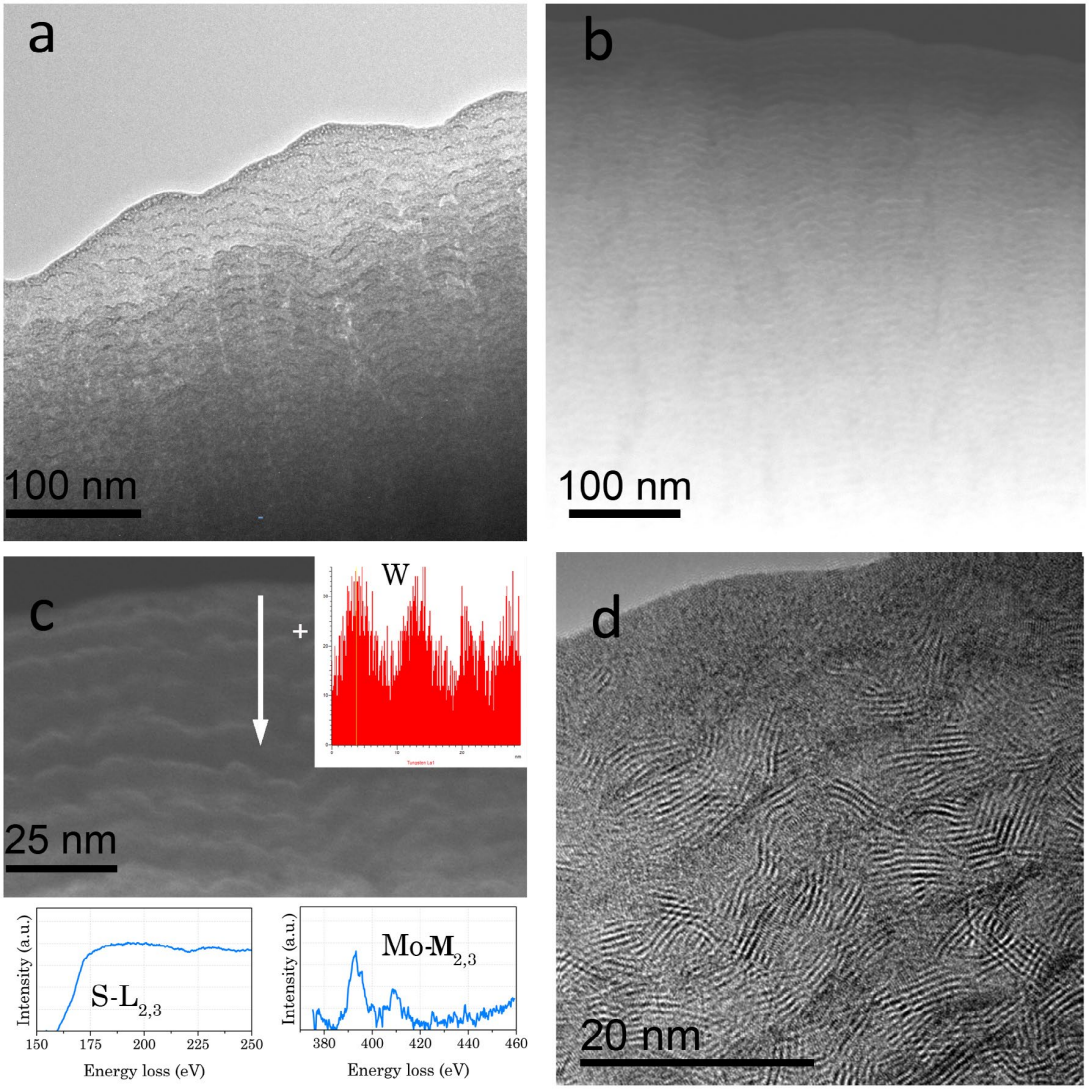
(a) TEM, (b, c) HAADF-STEM and (d) HRTEM micrographs of the MoS_2_-WC film. An EDX profile across the marked line and EELS spectra taken at the white cross are also shown in (c).

The advanced characterization of the nanostructure by TEM techniques has revealed some important differences in the samples. The MoS_2_ coating is formed by a homogeneous layer approximately 1.5 μm thick. A columnar nanostructure can be observed in the conventional TEM (Figure 2(a)) and HAADF-scanning TEM (STEM) images (Figure 2(b)). Small nanocracks are observed along the column boundaries. The inserted high resolution STEM-HAADF image reveals the presence of crystalline domains approximately 4‒12 nm in size. The measured D-spacing between the lattice fringes are found to be from 6.2 to 7.1 Å in different HRTEM images. A representative HRTEM image is shown in Figure 2(c). These values are in agreement with the separation between the (002) basal planes of the layered MoS_2_ crystal structure (6.2 Å). Moreover, lattice fringes of 2.5, 2.2, 1.5 Å have also been measured that can be assigned to the (102), (103) and (110) family planes of the same phase. The increased separation of the basal planes with respect to the theoretical value can be due to the defective structures and metal vacancies produced during physical vapor deposition processes. EDX quantitative analysis performed in different locations confirmed the metal deficiency, with S/Mo values between 2.2 and 2.7. For the MoS_2_-WC sample, a multilayered nanostructure is observed in the TEM and HAADF-STEM images (Figure 3(a) and (b), respectively). These layers are formed as a consequence of the rotation of the substrate and the sequential exposure of the two different targets. In the Z-contrast image of Figure 3(c) (Z is the atomic number), thicknesses of ~1 nm and ~6 nm have been measured for the brighter and darker layers, respectively. The EDX line profile obtained along these layers (see inset of Figure 3(c)) shows a W-enrichment of the brightest layers. The EELS spectra (S-L_2,3_ and Mo-M_2,3_ edges) measured on the marked point of the dark layer also confirm the presence of sulfur and molybdenum. In the HRTEM image (Figure 3(d)), some crystalline domains are observed between the W-rich layers. The measured d-spacing of the lattice fringes (~6.5 Å) can be assigned to the h-MoS_2_ phase. The size of these domains is approximately 3‒4 nm (five or six platelets), smaller than what is observed for sample MoS_2_. A d-spacing of ~2.3 Å is also measured in the W-rich layers corresponding to the (002) family plane of the h-W_2_C phase, but in general these layers seem quite amorphous.

**Figure 4.**
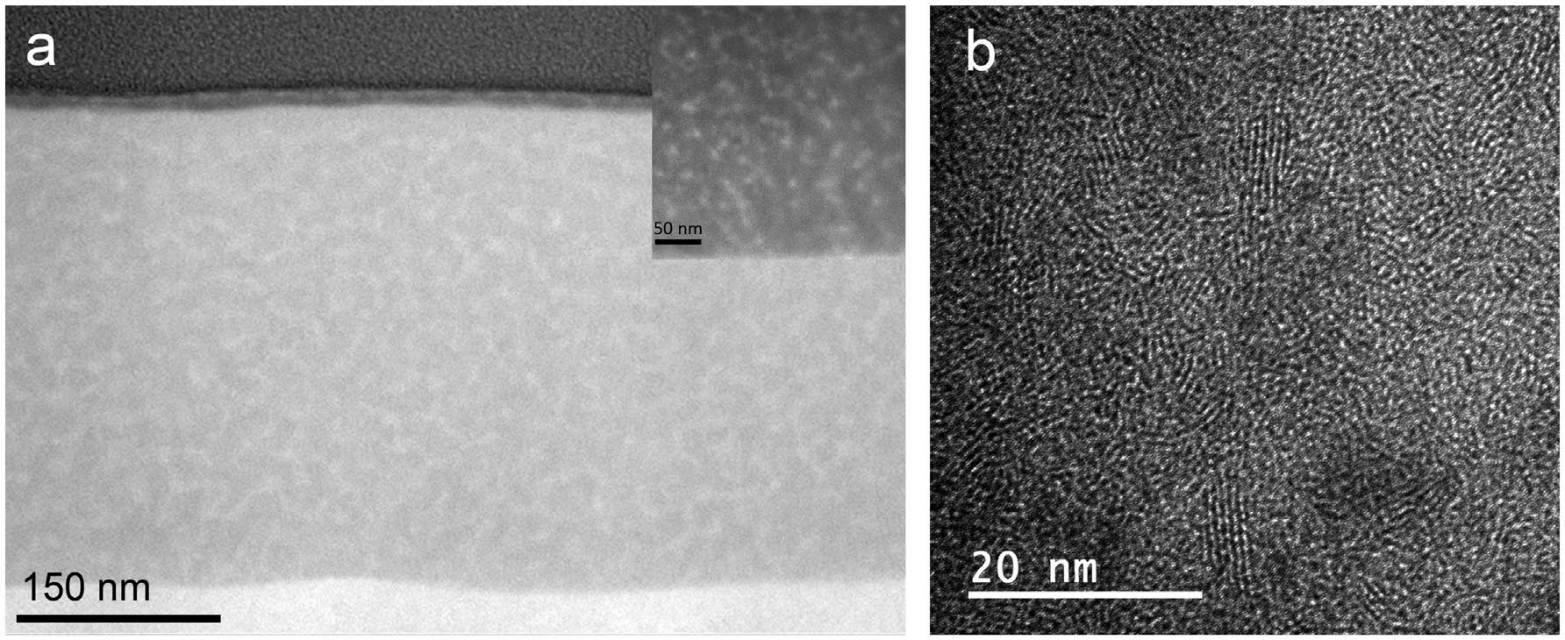
Z-contrast image of the topmost layer including an inset at higher magnification; and (b) HRTEM image of the WSe_x_ coating.

**Figure 5.**
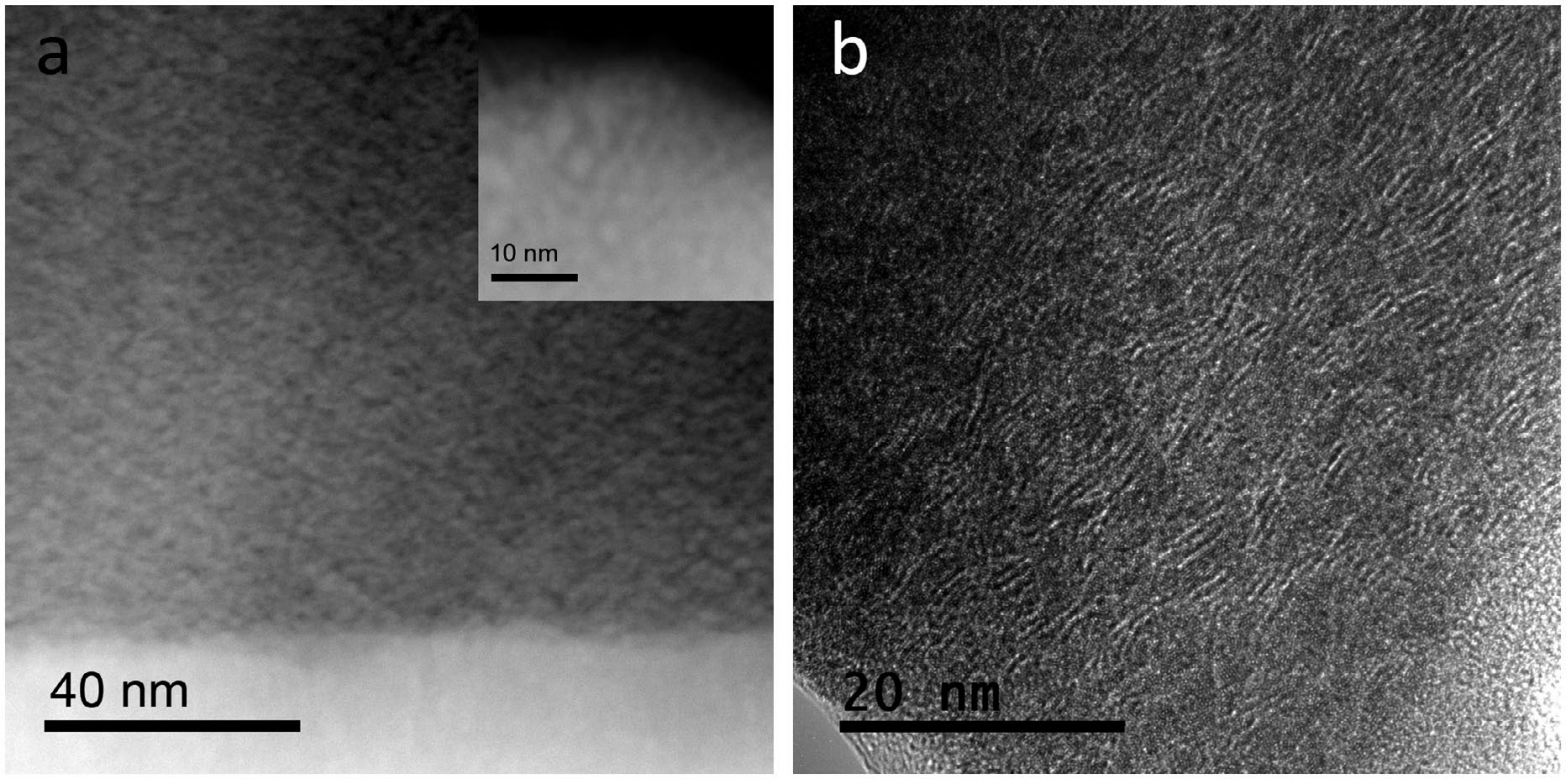
HAADF-STEM cross section showing an inset at higher magnification of the outermost part and (c) HRTEM micrograph of the WSe_x_-WC coating.

An exhaustive TEM characterization of the WSe_x_ sample has already been reported [18]. Three main regions with different Se/W ratio were clearly distinguishable: a bottom layer, mainly composed of metallic W, and two selenium-containing layers where the Se content stepwise increases between the second and third regions, reaching a maximum in the top layer. Figure 4(a) displays a higher magnification Z-contrast image of the uppermost region. Grains of 6‒10 nm size with higher intensity are embedded in a matrix of uniform contrast. The correlation between the HRTEM, HAADF-STEM and EDX analyses allows a conclusion that this uppermost region is formed by a nanocomposite of nonstoichiometric (Se deficient) nanocrystalline WSe_2_ surrounded by amorphous W(Se) grains, a-W(Se). Compared with the MoS_2_ coating, the WSe_x_ coating revealed a denser microstructure, with no clear evidence of nanocrack formation, but with a lower density of crystalline domains, as can be seen from Figures 2(c) and 4(b). The sizes of the crystalline domains of WSe_2_ and MoS_2_ are approximately 3‒6 nm and 4‒12 nm, respectively. These aspects help to explain the difference in the observed performance, as will be discussed later. Some small changes are produced when WC is added to the WSe_x_ coating. First, a bilayer structure is formed with the bottom layer richer in W (Se/W = 0.08) (cf. Figure 5(a)). Second, a polycrystalline character is noted, and some W_2_C crystals have been detected by HRTEM (not shown in Figure 5). The outermost layer presents the same nanocomposite structure (inset in Figure 5(a)) as the WSe_x_ but with a higher density of W-rich grains attributed to the higher W content (Se/W ratios of 0.45 and 1‒1.2 were obtained by EDX for WC-WSe_x_ and WSe_x_ respectively). The size of the lamellar structures also decreased (1.2–2 nm) with respect to pure WSe_x_. Figure 5(b) depicts a representative HRTEM image where a high density of lattices fringes is observed. D-spacings of 7.5, 2.4 and 2.1 Å have been measured in different micrographs, which can be assigned to the hexagonal WSe_x_ phase. A D-spacing of 2.2 Å can also be found and can be ascribed to the presence of W_2_C and W small crystals, as previously reported for WSe_x_.

**Figure 6.**
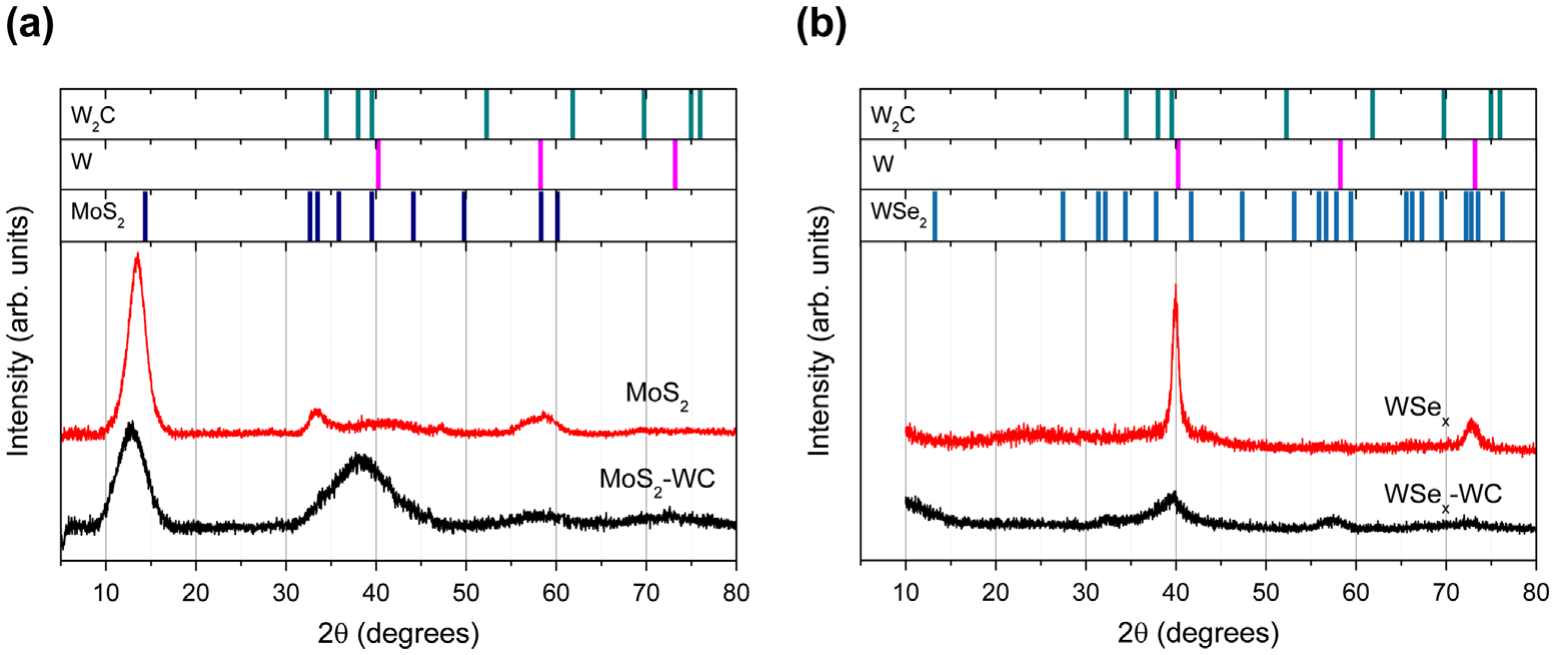
XRD diffractograms of the MoS_2_ (a) and WSe_2_-based (b) coatings.

The XRD diffractograms of the four samples under analysis are shown in Figure 6(a) and (b) for the disulfide and diselenide-based coatings, respectively. The position of the main diffraction peaks of W_2_C (PDF# 00-035-07), W (PDF # 4-806), MoS_2_-2H (PDF # 00-37-1492) and WSe_2_ (PDF # 38-1388) are included as references. In the first type, the most prominent feature is an intense peak at 13.4° (2θ°) that corresponds to the (002) reflection of the hexagonal MoS_2_. This particular structure has two S-Mo-S layers per unit cell in hexagonal symmetry with a separation of 6.2 Å. Compared with the MoS_2_ reference, a displacement of the (002) peak of ~1° and 1.5° towards lower angles is noticed for MoS_2_ and MoS_2_-WC, respectively. This fact confirms the increased separation between the Mo-S-Mo basal planes that was previously found by HRTEM. A reduction of the crystalline domain size is observed in the chalcogenide phases when co-sputtered with the WC target. In the pattern for the MoS_2_-WC sample, the main peak is due to the (002) basal planes, with a decreased intensity and a marked broadening with respect to the MoS_2_ single film. This agrees with the previous HRTEM results where the dimensions of the ordered regions were reduced from 4–12 to 3–4 nm. The broad band extending from 30 to 45° can be assigned to the overlapping W_2_C and MoS_2_ reflections. The prominent peaks in the WSe_x_ film at 39.9° and 73.1° correspond to metallic W, whereas the WSe_2_ reflections are not clearly detected. In the WSe_x_-WC coating, the intensities of the W peaks are strongly reduced, and a very broad band centered at 57.5° can be attributed to different families of planes of the WSe_2_ and W phases of reduced crystallite size and reduced crystallinity.

**Figure 7.**
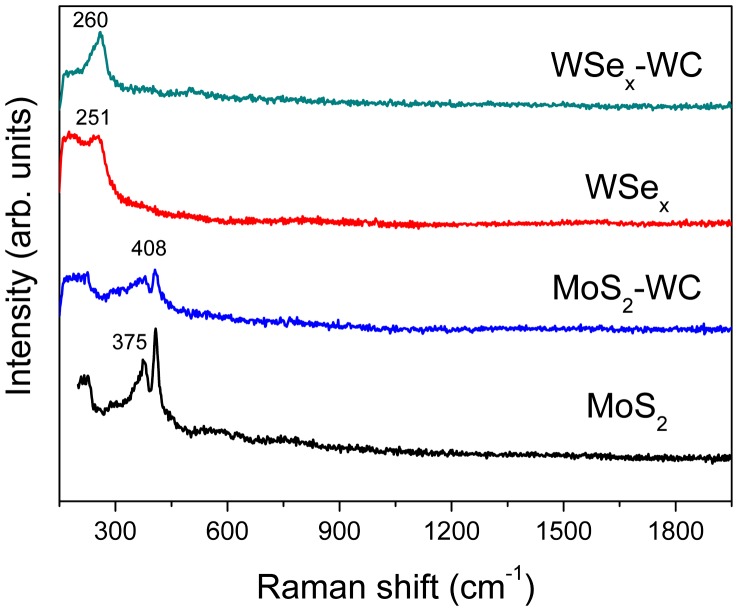
Raman spectra of the MoS_2_ and WSe_2_-based coatings.

The Raman spectra of the coatings are shown in Figure 7. The films based on MoS_2_ show a characteristic doublet formed by two peaks at 375 and 408 cm^−1^ [40]. The incorporation of the WC led to a decrease of the intensity of both peaks, indicative of a higher bond and angle disorder in the structure. This result agrees with HRTEM observations, where a decrease in the crystalline domains was observed. In the case of the tungsten selenides, the most characteristic feature is a narrow peak measured at approximately 260 cm^−1^ that develops more in the case of the WSe_x_-WC film [17].

### 3.2. Tribological characterization

**Figure 8.**
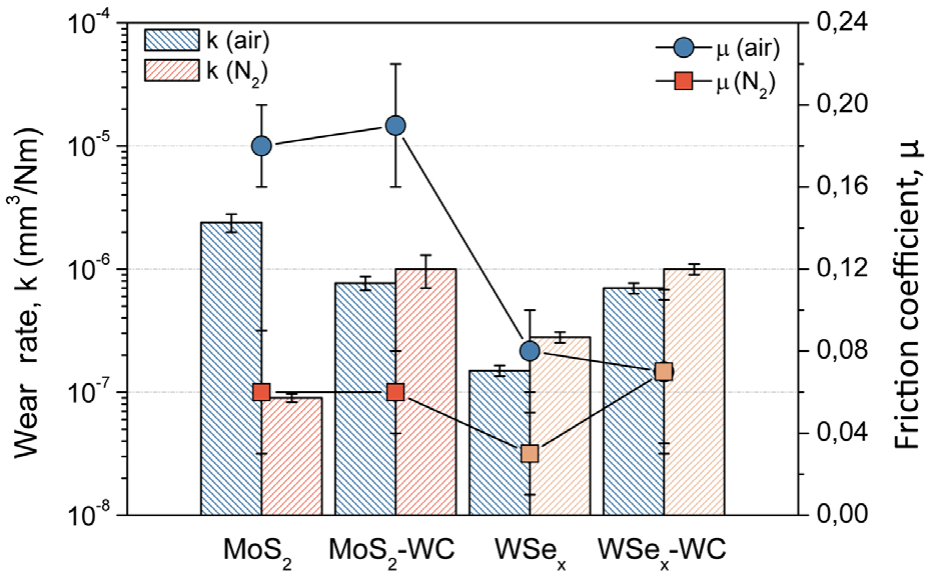
Tribological properties (mean friction coefficient and wear resistance) in ambient air (30–40% RH) and dry nitrogen (<7%) for the MoS_2_ and WSe_2_-based coatings.

The tribological behavior of the four coatings was compared in ambient air (30–40% relative humidity, RH) and dry nitrogen (<7% RH) using reciprocating sliding motion. The average friction coefficient and estimated wear rates are depicted in Figure 8. The influence of the surrounding environment is more evident in the friction coefficient value. As commented previously, the main disadvantage of these materials is their moisture sensitivity. For the molybdenum sulfide-based coatings, the values of the friction coefficient increased from typical values of 0.05–0.06 to 0.19. However, the tungsten selenides exhibit low friction coefficients even in the humid atmosphere (0.07–0.08). The higher chemical stability and decreased moisture sensitivity have already been demonstrated ^[17], [18]^ for this family of coatings [17,18] and other selenides [14,15]. It is also possible that the use of W instead of Mo contributes favorably, as WO_3_ is slightly more protective than MoO_3_ and provides lower friction in air (0.2–0.3 vs. 0.5–0.6, respectively) [41].

The pure MoS_2_ film exhibits the worst and best wear rate in ambient air (2.4 × 10^−6^ mm^3^ N^–1^m^–1^) and dry nitrogen (9 × 10^−8^ mm^3^ N^–1^m^–1^), respectively. As expected, the higher moisture sensitivity of the tribological behavior of MoS_2_ vs. the selenides and the excellent lubricity provided by the interlaminar structure are found. Among the different candidates, the pure WSe_x_ stands out for its good compromise of wear resistance and friction coefficient both in ambient air and in inert environments. The tribological behavior remains almost unaltered, showing similar friction coefficients (0.04–0.07) and wear rates (1.5–3 × 10^−7^ mm^3^ N^–1^m^–1^), independent of the nature of the environment.

**Figure 9.**
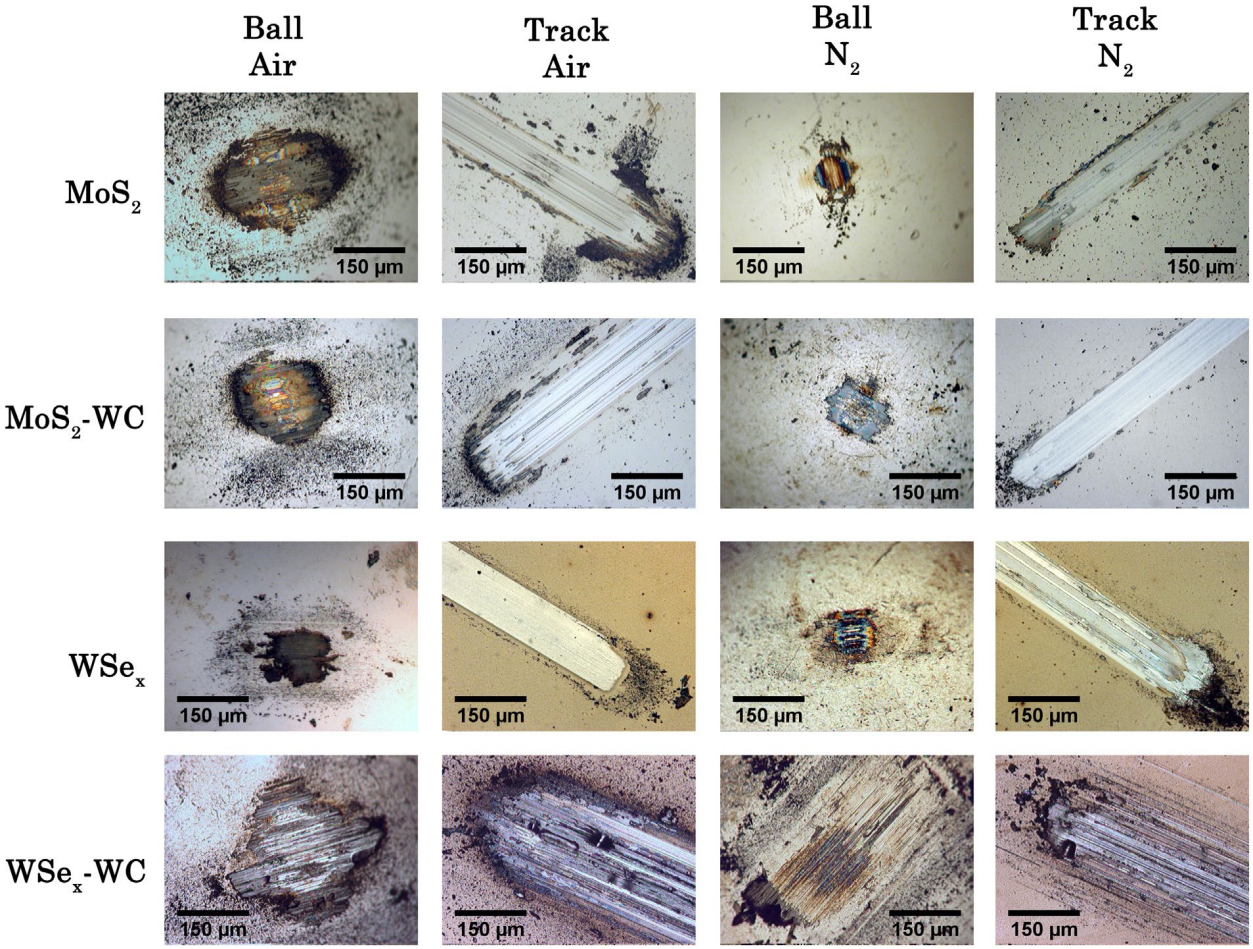
Optical micrographs taken from the ball and film wear counterfaces after the tribological tests run in ambient air and dry nitrogen for the films under study.

Both counterfaces (ball and film track) for the different coatings were inspected to obtain information about the friction and wear mechanism. Figure 9 depicts the optical micrographs taken in each case as a function of the type of atmosphere. The number of generated debris particles and the amount of material adhered to the ball is significantly higher under ambient air. The presence of water and oxygen molecules contributes to the oxidation, detachment of debris particles and film wear. The formation of a transfer film favors easy shearing by interfilm sliding, decreasing the friction coefficient, as observed previously in many MoS_2_ and DLC films [5,11,42]. The low shear strength across the basal planes serves to accommodate the relative motion. In addition, if the films are thick enough, the velocity can be accommodated as well by plastic deformation of this transferred layer. In this respect, the term ‘tribofilm’ seems more appropriate than ‘transfer film’, since the structural chemistry could be very different from that of the original films due to the combined action of friction and surrounding atmosphere.

When a nitrogen atmosphere is employed, the size of the tribolayer is reduced and concentrates in the contact region. The wear tracks appear smoother and free of particles at the edges but the presence of a heterogeneous third body film is observed inside. Under dry nitrogen, the third body particles replenish the track, forming a tribolayer that enables the easy shear and accommodates the motion as we showed in [17]. The wear mechanism in these conditions is purely abrasive (not a mixture of chemical and mechanical) and the film is gradually worn as the steel ball slides gently in successive cycles over the track. The track formed in the WSe_x_/WC sample exhibits larger dimensions and grooves inside. In this case, the introduction of the hard carbide phases favored the abrasive wear of the film. Moreover, the tribofilm formed on the ball is much thinner, likely due to the partial removal of the film by the hard debris particles generated in the contact.

**Figure 10.**
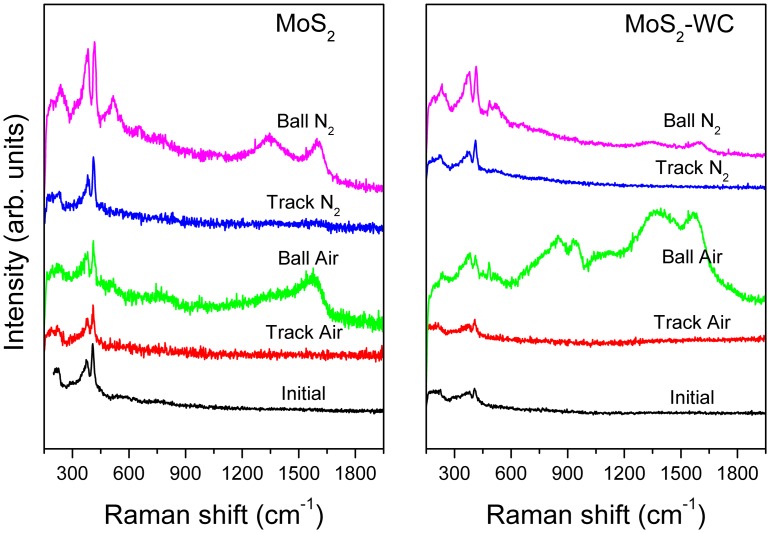
Raman spectra of the worn surfaces (material transferred to the ball and film track) in ambient air and dry nitrogen environments for the MoS_2_-based films.

With the aim of obtaining chemical and structural information about the changes induced by friction, a Raman analysis was carried out on the contact surfaces. In the next two figures, the spectra obtained from the counterfaces exposed to different chemical environments are presented. Figure 10 depicts the modifications observed with the molybdenum disulfides (MoS_2_ and MoS_2_-WC) coatings. The most significant variation is the increased intensity of the peaks at 375 and 408 cm^−1^, which is indicative of a structural ordering of the lamellar structure of MoS_2_. This is particularly significant in the nitrogen atmosphere and in the ball scar where the adhered material underwent mechanical deformation during the continuous passes. The formation of molybdenum oxides is denoted by the presence of a broad band between 600 and 900 cm^−1^ when the tests are performed in air. This oxidation phenomenon is more accentuated in the spectra obtained from the material stuck on the ball and MoS_2_-WC. The debris particles react with the oxygen molecules by a combined effect of friction and air exposure. Another feature of particular interest is the development of the D and G bands, characteristic of sp^2^-bonded carbon structures in the material adhered to the ball and the debris particles. Again, the presence of carbon is more accentuated in air than in dry nitrogen. A similar chemical segregation of carbon was highlighted by Colas et al. [13] in MoS_2_ coatings under air and dry nitrogen, which helped the lubrication efficiency. Apparently, the adventitious carbon present in the film concentrates in the third body material where it is subjected to pressure and friction forces during the circulation in the contact. This produces an increased order of the sp^2^-bonded carbon structures. Finally, these particles must be ejected out of the track or transferred to the pin since the spectra of the wear tracks are free of these bands.

**Figure 11.**
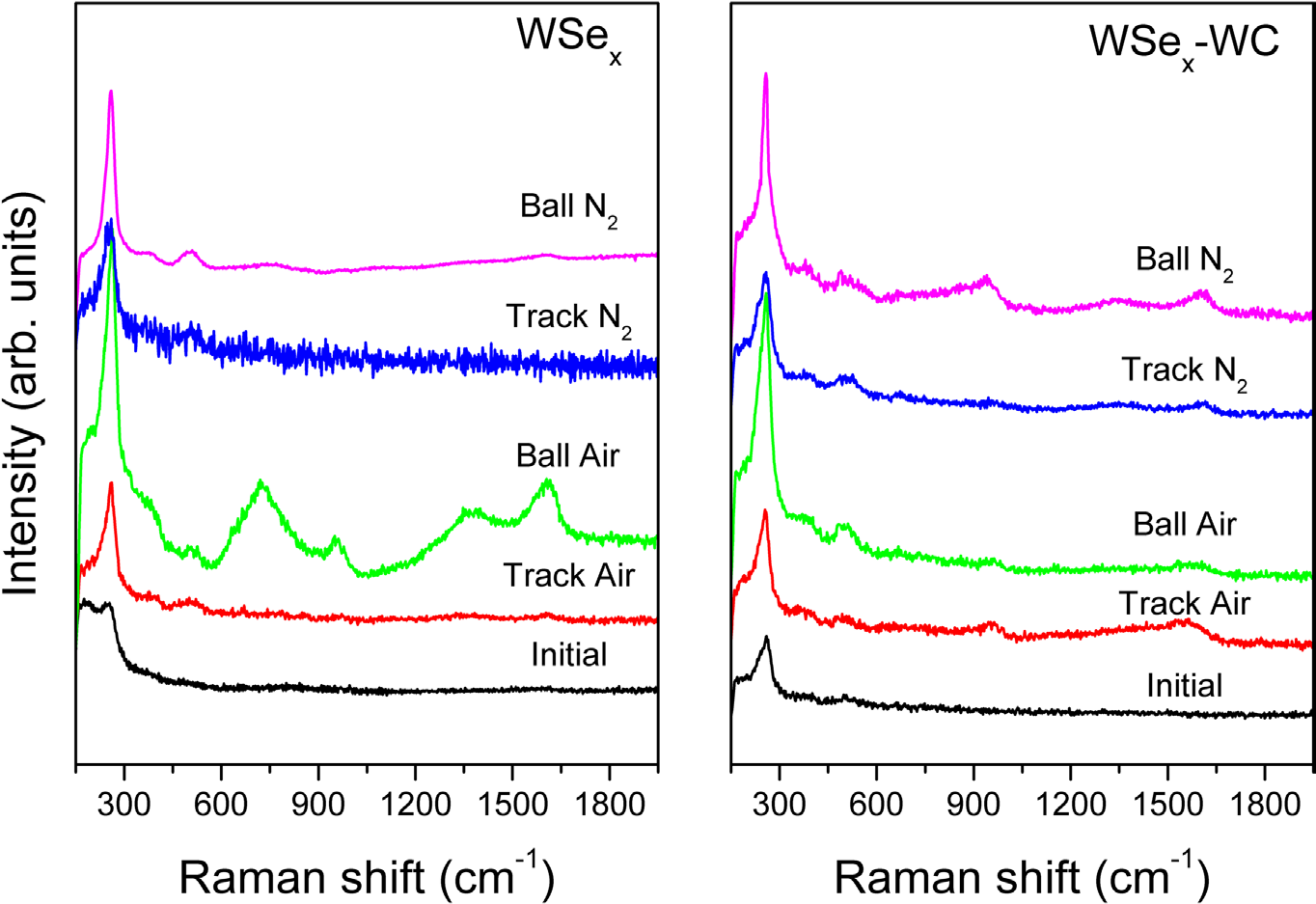
Raman spectra of the worn surfaces (material transferred to the ball and film track) in ambient air and dry nitrogen environments for the WSe_2_-based films.

Figure 11 shows the Raman spectra for the case of the tungsten selenides (WSe_x_ and WSe_x_-WC). The conclusions are rather similar in both cases and repeat those for the sulfides. A significant increase in the intensity of the 260 cm^−1^ peak is observed after friction in all cases. This enhancement, together with the associated bands at 375 and 515 cm^−1^, are due to the WSe_2_ nanocrystals. This phenomenon has been previously demonstrated by transmission electron microscopy performed on the contact region. An increased order and orientation of the basal planes of WSe_2_ are observed parallel to the wear scar surface [18]. The existence of new bands due to the oxidation of the tungsten selenide is sometimes observed. Hence, a broad band from 600 to 800 cm^−1^ and a second peak close to 960 cm^−1^ are assigned to W-O-W stretching bonds and W=O bonds, respectively [43]. In a similar manner as the MoS_2_ coatings, carbon segregates and appears concentrated in the material adhered to the ball as a component of the third body layer. Comparatively, the tungsten selenides showed a higher enhancement of the bands related to the W-Se bonds than the molybdenum sulfides, particularly in the case of ambient air. This would account for the improved performance of this type of coating in this environment where friction did not increase as much as in the case of MoS_2_. The differences in nanostructure and chemical reactivity can support the observed differences in the friction coefficient in ambient air. The more columnar structure of the MoS_2_ coatings and the inferior chemical resistance allow the oxygen to diffuse into the film, making molybdenum oxidation easier; whereas the lower chemical sensitivity of the WSe_x_-based coatings can be explained by their denser nanocomposite nanostructure and the presence of W-rich domains, as highlighted in our previous study [18].

**Figure 12.**
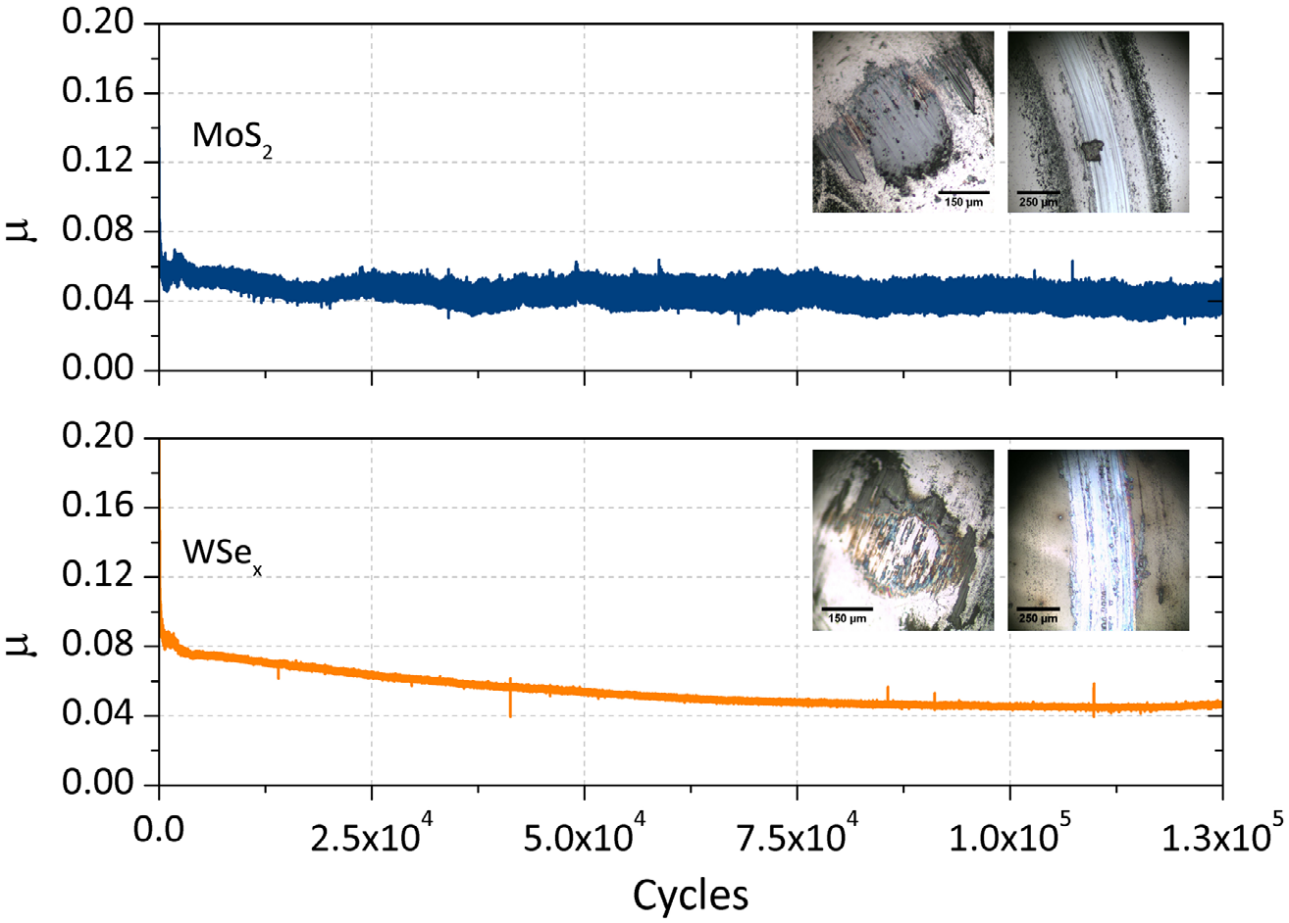
Long duration friction tests in dry nitrogen for the MoS_2_ and WSe_x_ films.

The two samples exhibiting the best behavior in the dry nitrogen atmosphere (MoS_2_ and WSe_x_) were selected for further structural analysis and tribological testing. These samples were then tested in long duration tests for 125,000 cycles using a rotating ball-on-disk tribometer in dry nitrogen. Figure 12 shows the friction curves for both coatings. The achievement of a low friction steady-state at 0.03–0.05 in both cases proved their excellent performance in an inert environment. The measurement of the wear resistance for these two cases found values of 9 × 10^−8^ mm^3^ N^–1^m^–1^ (MoS_2_) and 2 × 10^−8^ mm^3^ N^–1^m^–1^ (WSe_x_), indicating that WSe_x_ has a better wear resistance. In the WSe_x_ film, the architecture and chemical composition was tailored to satisfy requirements for load bearing capacity, mechanical support and lubricity, provided by the combination of soft and lubricant phases. This nanodesign allows an improved endurance when subjecting the coating to longer friction tests. Such solid lubricant coatings with low friction either in ambient or inert atmospheres are thus very interesting for space applications whose components are exposed to different surrounding atmospheres from their preparation conditions until reaching the orbit.

## 4. Conclusions

The lubricating performance of layered transition metal chalcogenides thin films (MoS_2_ and WSe_2_) and their corresponding combinations with WC phase prepared by magnetron sputtering was comparatively studied using the same tribological testing conditions. The WSe_x_-based films can provide approximately the same low friction coefficient and wear rate in ambient air and dry nitrogen, an improvement over the tribological performance of the MoS_2_ coatings. However, reinforcement of the wear resistance by inclusion of the WC phase is only significant for MoS_2_ tested in air. The MoS_2_-WC and WSe_x_-WC coatings exhibited smaller crystalline domain sizes and lower crystallinity. The analysis of the contact surfaces by Raman and optical microscopy provided evidence of the segregation of carbon and the formation of metallic oxides in the third body material developed in the pin, together with an increased ordering of the lamellar structure. In the wear tracks, only increased order was detected with respect to the original coating, which occurs to a higher extent in the WSe_x_-based films, consistent with the improvement in the tribological behavior. In addition to the higher moisture resistance of the metal selenides, the investigated WSe_x_ coatings contain W-rich areas that contribute to capturing the oxygen, forming tungsten oxides and preserving the lubricant WSe_2_ phase.

Comparison under dry nitrogen conditions of the best coating of each type (MoS_2_ and WSe_x_) revealed a lower wear rate for WSe_x_ (2 × 10^−8^ mm^3^ N^–1^m^–1^ for 125,000 cycles). This enhanced performance makes this nanocomposite material a good competitor for MoS_2_ as a solid lubricant for vacuum and space applications where lubricated components are exposed to variable surrounding environments.

## Disclosure statement

No potential conflict of interest was reported by the authors.

## Funding

The Spanish Ministry of Economy, Industry and Competitiveness [projects n° MAT2010-21597-C02-01, MAT2011-29074-C02-01; MAT2015-65539-P; MAT2015-69035-REDC], Junta de Andalucía [P10-TEP-67182] and Spanish National Research Council (CSIC) [201560E013] are acknowledged for their financial support. Lamellae preparation was conducted in the ‘Laboratorio de Microscopias Avanzadas’ at ‘Instituto de Nanociencia de Aragón-Universidad de Zaragoza’. Authors acknowledge the LMA-INA for offering access to their instruments and expertise.
